# Supplementary figures and images for: Human genetic evidence enriched for side effects of approved drugs
Source: PLoS Genet. 2025 Mar 31;21(3):e1011638. doi: 10.1371/journal.pgen.1011638 (PMC11977994; doi:10.1371/journal.pgen.1011638)

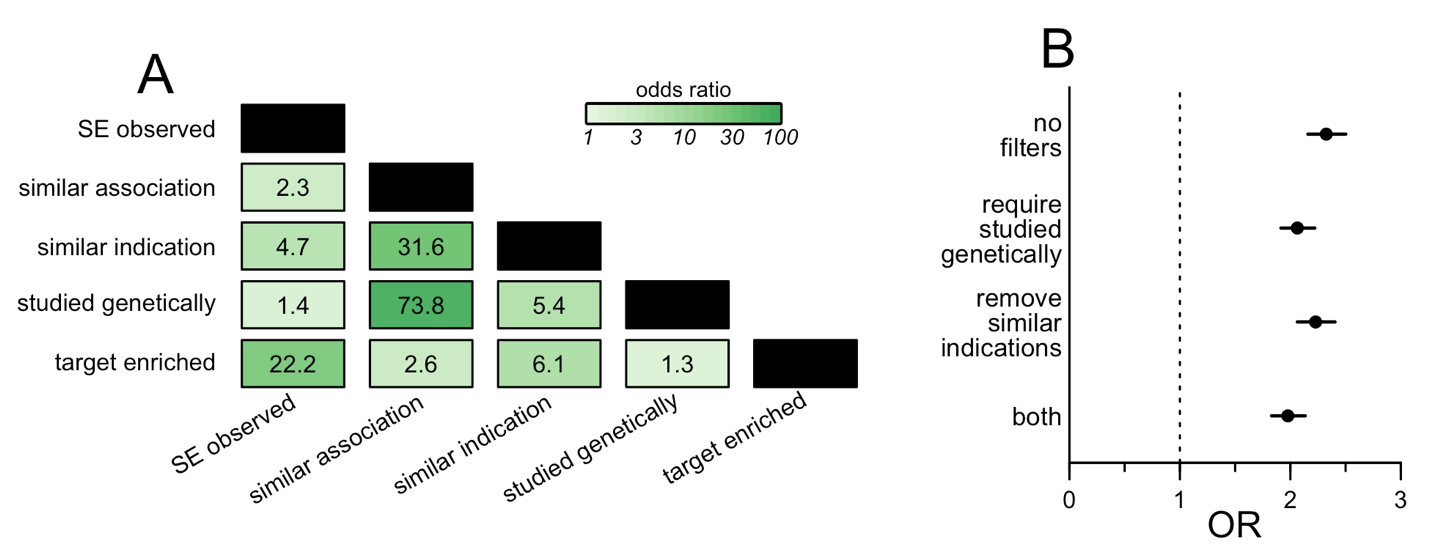

Supplement: S1 Fig — A) Correlogram showing the odds ratios (ORs) by Fisher exact test for enrichment of all combinations of properties (S1 Table and Methods) evaluated in the dataset. B) OR for enrichment of genetic evidence vs. SE observed, with the indicated filters applied. (TIFF) [file pgen.1011638.s001.tiff]

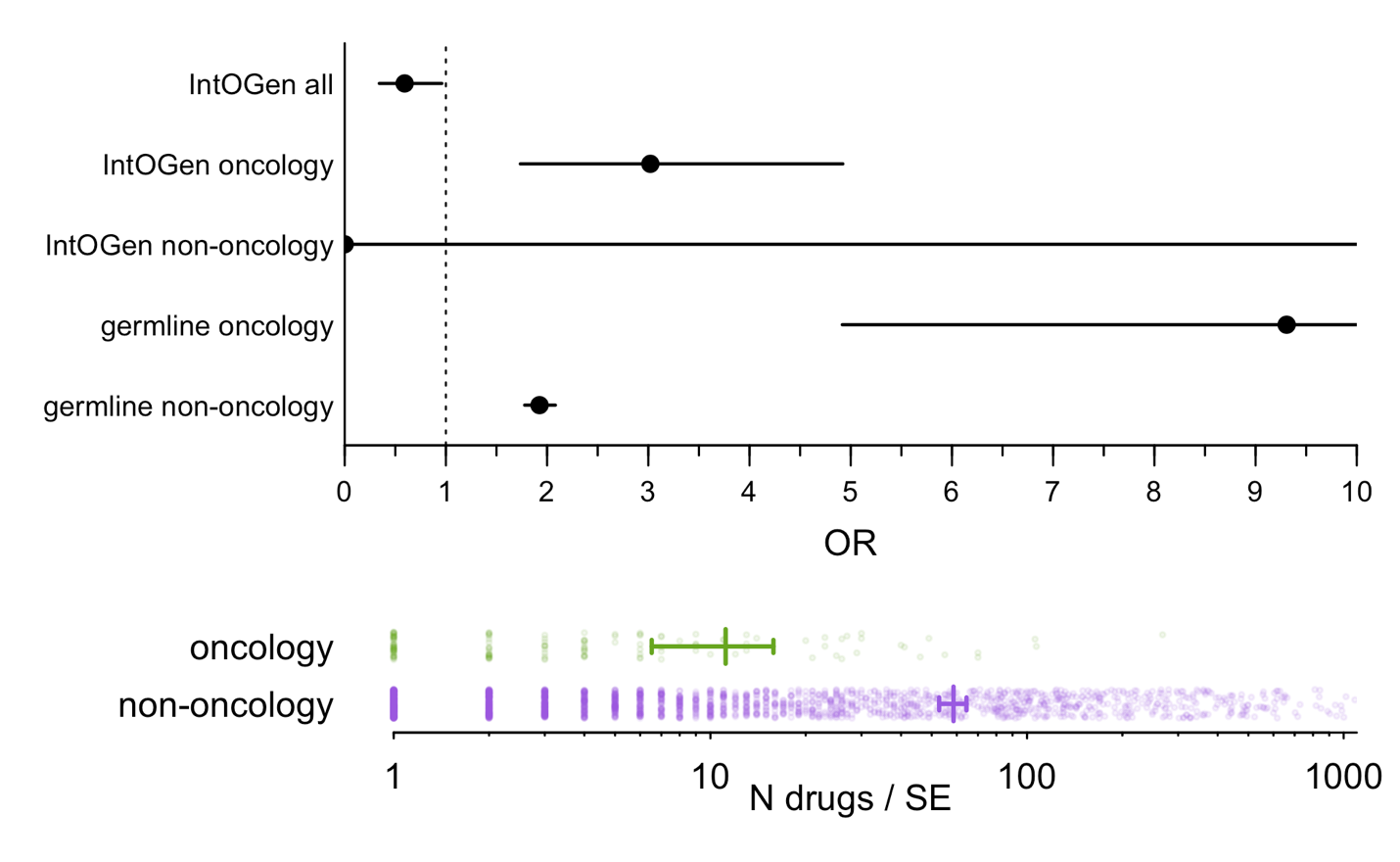

Supplement: S2 Fig — A) Forest plot of OR by source of evidence (IntOGen somatic evidence vs. all sources of germline evidence) versus oncological and non-oncological SEs. B) Drug specificity of oncological and non-oncological SEs. IntOGen overall has an OR < 1 because its somatic evidence are almost exclusively similar to oncological SEs, which are more drug-specific than non-oncological SEs. Thus, the IntoGen OR for oncology only is shown in Fig 1. Germline evidence appears to have a higher OR than somatic evidence for oncology. Note that the germline evidence for oncology is driven by GWAS associations for X genes: CYP19A1 (endometrial neoplasms), ESR1 (breast neoplasms), FGFR2 (neoplasms), INSR (polycystic ovary syndrome), and SRD5A2 (breast neoplasms). (TIFF) [file pgen.1011638.s002.tiff]

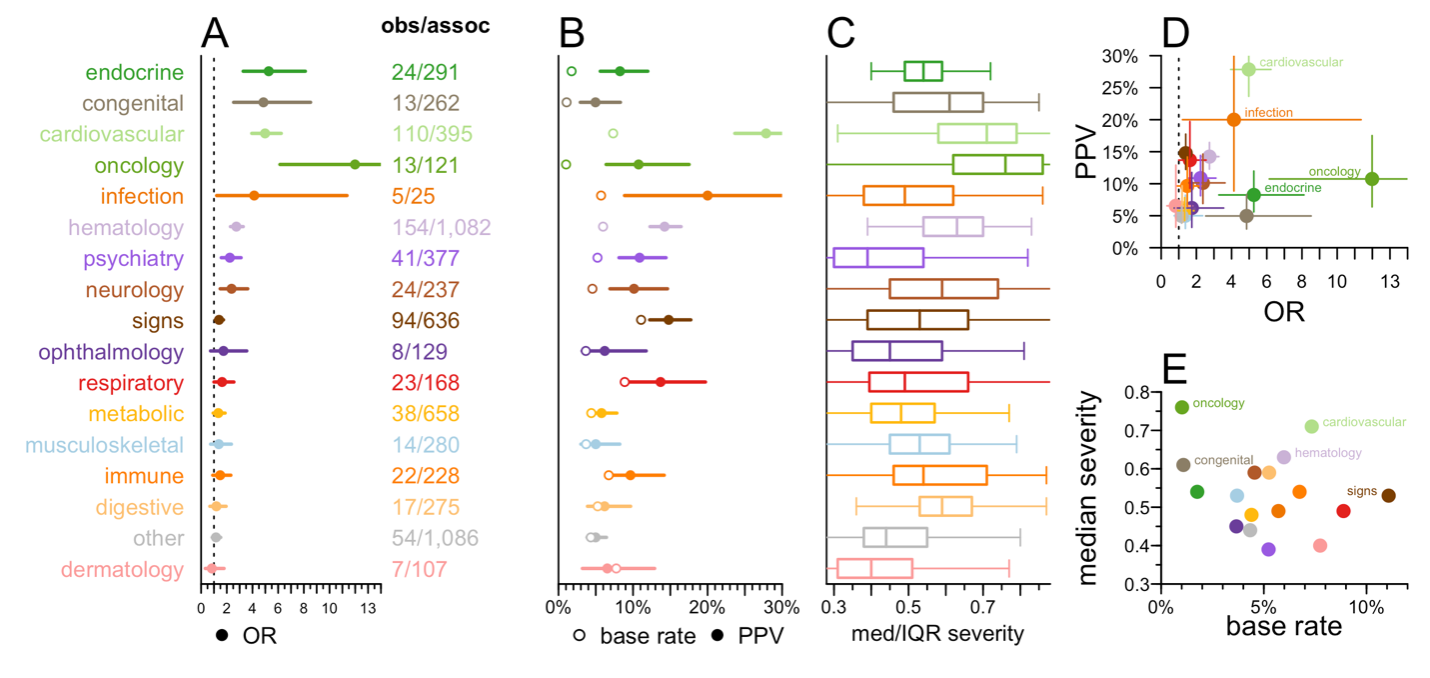

Supplement: S3 Fig — As Fig 4, but within each MeSH area, any drug with any indication in that area is removed. (TIFF) [file pgen.1011638.s003.tiff]
