# Supplementary material for: Human genetic evidence enriched for side effects of approved drugs
Source: PLoS Genet. 2025 Mar 31;21(3):e1011638. doi: 10.1371/journal.pgen.1011638 (PMC11977994; doi:10.1371/journal.pgen.1011638)
Supplement: Attachment — Submitted filename: GeneticSafetyRisk_R2R-PLoS_Sub2.docx [file pgen.1011638.s006.docx]

You are being carbon copied ("cc:'d") on an e-mail "To" "Matthew R Nelson" mnelson@genscience.com

CC: "Eric Vallabh Minikel" ericminikel@gmail.com

Dear Dr Nelson,

Thank you very much for submitting your Research Article entitled 'Human genetic evidence enriched for side effects of approved drugs' to PLOS Genetics.

The manuscript was fully evaluated at the editorial level and by independent peer reviewers. The reviewers appreciated the attention to an important topic but identified some concerns that we ask you address in a revised manuscript.

We therefore ask you to modify the manuscript according to the review recommendations. Your revisions should address the specific points made by each reviewer.

In addition we ask that you:

1) Provide a detailed list of your responses to the review comments and a description of the changes you have made in the manuscript.

2) Upload a Striking Image with a corresponding caption to accompany your manuscript if one is available (either a new image or an existing one from within your manuscript). If this image is judged to be suitable, it may be featured on our website. Images should ideally be high resolution, eye-catching, single panel square images. For examples, please browse our archive. If your image is from someone other than yourself, please ensure that the artist has read and agreed to the terms and conditions of the Creative Commons Attribution License. Note: we cannot publish copyrighted images.

We hope to receive your revised manuscript within the next 30 days. If you anticipate any delay in its return, we would ask you to let us know the expected resubmission date by email to plosgenetics@plos.org.

If present, accompanying reviewer attachments should be included with this email; please notify the journal office if any appear to be missing. They will also be available for download from the link below. You can use this link to log into the system when you are ready to submit a revised version, having first consulted our Submission Checklist.

While revising your submission, please upload your figure files to the Preflight Analysis and Conversion Engine (PACE) digital diagnostic tool. PACE helps ensure that figures meet PLOS requirements. To use PACE, you must first register as a user. Then, login and navigate to the UPLOAD tab, where you will find detailed instructions on how to use the tool. If you encounter any issues or have any questions when using PACE, please email us at figures@plos.org.

Please be aware that our data availability policy requires that all numerical data underlying graphs or summary statistics are included with the submission, and you will need to provide this upon resubmission if not already present. In addition, we do not permit the inclusion of phrases such as "data not shown" or "unpublished results" in manuscripts. All points should be backed up by data provided with the submission.

To enhance the reproducibility of your results, we recommend that you deposit your laboratory protocols in protocols.io, where a protocol can be assigned its own identifier (DOI) such that it can be cited independently in the future. Additionally, PLOS ONE offers an option to publish peer-reviewed clinical study protocols. Read more information on sharing protocols at https://plos.org/protocols?utm_medium=editorial-email&utm_source=authorletters&utm_campaign=protocols

Please review your reference list to ensure that it is complete and correct. If you have cited papers that have been retracted, please include the rationale for doing so in the manuscript text, or remove these references and replace them with relevant current references. Any changes to the reference list should be mentioned in the rebuttal letter that accompanies your revised manuscript. If you need to cite a retracted article, indicate the article’s retracted status in the References list and also include a citation and full reference for the retraction notice.

PLOS has incorporated Similarity Check, powered by iThenticate, into its journal-wide submission system in order to screen submitted content for originality before publication. Each PLOS journal undertakes screening on a proportion of submitted articles. You will be contacted if needed following the screening process.

To resubmit, log into your Editorial Manager account and select the option 'Revise Submission' in the 'Submissions Needing Revision' folder.

Please let us know if you have any questions while making these revisions.

Yours sincerely,

Lynn Petukhova, Ph.D.

Guest Editor

PLOS Genetics

Hua Tang

Section Editor

PLOS Genetics

Reviewer's Responses to Questions

Comments to the Authors:

Please note here if the review is uploaded as an attachment.

**Reviewer #1: Described in this manuscript is a detailed analysis of how human genetics can help predict the occurrence of drug side-effects (SEs). To that effect, the Authors used extensive databases on marketed drugs and their reported SEs, matching them with databases on the genetic basis of traits similar to the SEs. They observed that the risk of SE in presence of a genetic association between the drug target gene and latter trait was increased by 2-fold. From this observation, they suggest that incorporating human genetics could help predict future SEs for investigational drugs.**

**The topic is very timely and relevant, not only for the pharma industry (SEs are responsible for a substantial proportion of failures in development), but also for public health, considering that SEs are responsible for 5-10% of hospital admissions.**

**The work presented here is a logical extension of previous, outstanding work performed by the same Authors on the value of human genetics to predict the probability of success for a drug target to eventually go through the hurdles of drug development to regulatory approval.**

**The manuscript is well written. Methods are sound and sophisticated, yet well presented. Data is well presented and carefully interpreted, conclusions are solidly supported by the data and potential implications of the findings cautiously presented.**

We thank the reviewer for their careful read of our manuscript and thoughtful comments.

**In my opinion, the text is quite dry, and the manuscript would greatly benefit from specific examples that shall illustrate the concept, and the type of analyses performed. In particular, Readers would appreciate to see the name of a drug, the type of ADR, traits similar to SEs, examples of SEs which are very rare to common, etc…**

We have added several specific examples throughout the Results.

**The description of PPVs is highly relevant, although, as discussed by the Authors, they pertain here to approved drugs and may not necessarily apply to drugs in development. In my opinion, it may be worthwhile to emphasize the fact that PPVs are hardly better than base rate (i.e. not informative) for SEs in specific functions/organs.**

Added: “In contrast, several SE areas exhibited ORs barely greater than 1, and accordingly had PPV barely above their low base rates (Figure 4A-B).”

**PPVs clearly stand out for cardiovascular SEs. This is somehow surprising considering that PPVs are much lower for metabolic traits. A deep dive into this specific type of SEs would be appreciated.**

We have added new supplementary tables (S25 and S26) showing the OR, PPV, and NPV for each specific genetic association and each specific SE, which will allow the reader to dig into specific SEs of interest.

**A discussion on NPV would be appreciated, i.e. how predictive for SEs is the absence of any genetic association ? Maybe this cannot be analyzed.**

The new supplementary tables include NPV. We have also added a brief mention in the text: “Negative predictive values (NPV) was generally high across all SE areas (range 88-99%; Table S23).” From spending some time looking at different NPVs across the slices of data, it is not clear to us that the NPV is all that useful — mostly it is just high when the side effect is rare to begin with and no one developing a drug would be concerned in the first place. But the data are there in case someone else wants to dig in, and it is certainly possible that there is more to unpack there than we have appreciated.

**The absence of correlation between base rate and severity is intuitively surprising, considering the fact that certain drugs would be approved with severe SEs, as long as these SEs are very rare. Any explanation to Figure 4E ?**

We have clarified that there is some correlation observed: “In other words, the base rate was lowest for the most severe quartile of SEs (6.1%, versus 10.9% for the least severe quartile of drugs). (Drugs without a severity score assigned had the lowest base rate of all, perhaps simply reflecting that less commonly encountered side effects were less likely to be included in the survey used to rank severity).”

**Reviewer #2: Minikel & Nelson focus a different lens on the data & methods assembled for their recent publication (Minikel et al. Nature, 2024; Minikel et al. medRxiv, 2023), to assess the genotype:phenotype correlation of side effects as opposed to lead indication success in clinical trials. One thing that the authors don't discuss but I believe is important, is what was their null hypothesis here? Given their previous publications and that they filtered their data to enrich their analysis with side effect traits that have been genetically investigated, null was that you'd have positive OR. And in fact, in aggregate that's what they see with and OR ~2 of what Nelson published in 2015 for association of genetics with drug approval. Pharmacological perturbation recapitulating genetic associations should have been the null in my opinion, therefore the results follow the null and the results seen in previous investigations (ref. 7 & 8). Was the reason for this work to more fully test the results of ref. 8?**

**The potential impact of the results of this investigation is limited because the side effects investigated are of approved drugs, therefore their liability to a development program are minimal and would be unlikely to influence go/no-go decisions for programs. The results presented are also very high-level and very heterogeneous (Fig 4), therefore also difficult to draw any strong conclusions nor think about how to operationalize this information into any practice in drug development. To no fault of the authors, the data available to analyze for the study is almost 10 years old and therefore limits an ability to see if the trends identified here have changed at all since the publication of Nelson et al. Nature Genetics, 2015 which has influenced the number of targets entering drug development with human genetic evidence.**

We thank the reviewer for their careful read of our manuscript and thoughtful comments.

**Perhaps beyond the scope the authors intended for this work, but to me the most interesting (and potentially useful) questions have not been asked and important aspects of the data not discussed. I'll detail these critiques here:**

**1. I'd be interested to know the distribution of the primary indications for the approved drugs analyzed here, the distribution of year of approval, and the median number of SEs. Is there a skew in indications that might have a skewed risk:benefit ratio such that some indications can get approved with more and/or more severe side effects? Has the number of SEs for drugs changed over time of approval, for example do newer drugs have fewer SEs and older drugs? Does this information confound the results of these results at all?**

We thank the reviewer for this suggestion. While we are able to comply several of the requested new analyses below, unfortunately point #1 is difficult for us: we do not have year of approval for all of these drugs, and even when we do, it is per drug, not per drug-indication pair, such that we do not know which of multiple indications is the “primary.”

**2. Is the OR of the genetic association to SEs at all associated/correlated with whether there is genetic evidence for the target in the primary indication?**

Added a long paragraph of new analyses beginning “We wondered how the enrichment of genetic evidence for side effects interacts with genetic evidence for the drug’s indication….”

**3. Are the targets that are associated with SEs more pleiotropic (i.e. have more associations to different phenotypes) than the "average" (could be defined in many ways) gene?**

Added: “When we tested the sensitivity of OR to the SE-trait similarity threshold, we observed ORs at least slightly above 1 at all thresholds tested (minimum OR = 1.14). We wondered whether this might reflect that more pleiotropic targets, which have more associations, also have more side effects. Overall, however, there was no correlation between a drug’s count of observed side effects an the count of unique traits to which its target is genetically associated (rho = -0.01, P = 0.72, Pearson’s correlation).”

**4. Is the direction of effect between the pharmacological perturbation and the genetic signal consistent or inconsistent?**

**a) This is the biggest question that pops out to me from the manuscript. You state that you've removed SEs that are similar to the approved indication. How frequent was this? This is a very surprising result because it suggests that a single target has completely different effect directions on very similar traits.**

**b) Extension of question 3 but in terms of directionality. If the target for the approved indication is a pleiotropic target, then you'd expect the gene has to have different directions of effect on the approved indication and the SE phenotype. Is this in fact true on average or not?**

We have added a statistic to answer this question: “At the 0.9 similarity threshold selected here, only 334/45,475 (0.73%) observed drug-SE pairs were removed from analysis due to the similar indication filter.”

We have also edited the text to re-emphasize the fact that not all reported SEs are actually causally related to the drug: “Still other SEs are simply adverse events that get reported and may become enshrined in drug labels despite not being causally related to the drug at all: some may be coincidental, or may be associated with the indication for which the drug is being prescribed in the first place. “

And in response to B we have added to Discussion: “An additional limitation is that our analysis does not account for direction of effect, either of the drug (agonist vs. antagonist) or of the genetic association, due to a lack of annotations in the source databases we utilized.”

**5. Was there a difference between the modality of the approved drug (small molecule vs. antibody vs. etc.)? Potential proxy for the specificity of the approved molecule.**

**a) perhaps related question about specificity, what are the gene classes (kinases, receptors) of the targets whose SEs have genetic evidence. Are there close paralogs in the genome or highly similar genes with similar genetic associations?**

**b) For the analysis in Figure 3, you ignored whether the SE was from targeting the same gene. This seems like a missed opportunity to game more insight. If not the same gene, but part of the same protein complex or known protein-protein interaction that provides something that could be operationalized in drug development just like for efficacy determination.**

SIDER only captures data on 2 monoclonal antibody drugs (ofatumumab and ustekinumab), making the modality analysis impossible.

The question of whether side effects are shared across targets of the same class (e.g. kinases, GPCRs) is an interesting one that we feel is beyond the scope of the current paper, as this would require a significant new analytical framework.

As for part b, we don’t have a way to determine whether two instances of SEs arise from targeting the same gene, because we do not know which SEs are on-target effects versus off-target or incidental.

**6. Perhaps I just don't understand Figure 1, as the language used to describe what is being shown is a bit hard to follow. But I believe that Fig1A is showing the OR of genetic evidence for the reported SE as you vary the phenotypic similarity between the stated SE and the proxy phenotype you use to look for genetic evidence. Given this understanding this figure makes absolutely no intuitive sense to me. The first part of the curve is the expectation, the further the SE is from the "genetic trait" the lower the OR is and essentially is an OR ~1 at around a similarity of ~ 0.7. But then the curve increase as you approach similarity of 0.5 and hits a new local minimum at a similarity of 0.2. There is no exploration or discussion of this fact in the manuscript. Is this simply a power thing given the number of observations detailed on the x-axis. Or this this noise? How can you account for this?**

**a) Generally, I don't have a strong intuition for the trait similarity metric and how it behaves. Just because it is a value between 0 and 1 doesn't mean that its behavior is similar across the spectrum. How much of the results are because the performance of the metric? Is the difference between 0.2 and 0.5 the same between 0.5 and 0.8? How have you chosen thresholds here and how does that relate to attributes of the metric? How different would results be if you used HPO or some other phenotype ontology instead of MeSH?**

Added: “As described in the Introduction, given the strong prior evidence that human genetics can predict on-target pharmacology, we expected to see ORs greater than 1, and to reject the null hypothesis that genetic evidence does not enrich for observed side effects. Of greater interest is at what threshold of similarly the enrichment is observed. When we tested the sensitivity of OR to the SE-trait similarity threshold, we observed ORs at least slightly above 1 at all thresholds tested (minimum OR = 1.14)… the OR inflected at a similarity threshold of 0.75 and rose sharply thereafter, reaching OR=2.0 at a threshold of similarity 0.90. Thus, genetic evidence is most predictive of side effects that are extremely similar to the genetically associated trait.”

**7. Fig2 also a bit confusing. What is different about the "common" and "frequent" class? The fact that "frequent" has an OR ~1 while "common" has an OR ~3 makes no sense, especially given that the numerical frequency plot above shows a significant jump in OR as you get above 10% frequency.**

“Common” vs. “frequent”, etc. are not our own designations but simply reflected inconsistent terminology used by regulators on drug labels and thus captured in SIDER. To simplify this for the reader, we have combined “uncommon/infrequent” into one class and “common/frequent” into another and re-made the figure; this also simplifies the explanation of the statistical analysis in the Results text.

**8. Wouldn't you expect that the base rate of severe SEs (Fig3D) to be the lowest given that those SEs would make the risk:benefit ratio get to the point where regulators wouldn't approve the drug? What primary indications had the highest frequency of severe SEs?**

It is indeed the case that the base rate is lowest for most severe SEs, and we have added language to highlight this: “In other words, the base rate was lowest for the most severe quartile of SEs (6.1%, versus 10.9% for the least severe quartile of drugs). (Drugs without a severity score assigned had the lowest base rate of all, perhaps simply reflecting that less commonly encountered side effects were less likely to be included in the survey used to rank severity).”

**9. Perhaps I also don't understand Figure 4 because of the language used to describe what is being shown. But the description, "binned by the SE’s top-level heading within the Medical Subject Headings (MeSH) ontology.", says to me that the terms on the y-axis of the forest plot are the SEs. Which would mean, that there are approved drugs where the SE is cancer?!? What does it mean to have an SE that is congenital? Does this mean that if pregnant women are given the drug their babies have issues?**

Yes, the reviewer’s stated assumptions are correct. For cancer, there are 229 unique drugs with a total of 851 side effects reported that are mapped to the “cancer” section of the MeSH ontology. Of these 851, there are 288 instances where the indication is also cancer; these are removed in the “hard filter” mode in Figure S3. Bear in mind that the actual label may read warning something like “may increase your risk of cancer”, rather than “this drug causes cancer.” Whether all of these are truly causal associations is beyond the scope of this paper, but looking through the list there are clearly instances where the association is real, for instance, drugs targeting *ESR1* for hormone replacement therapy are associated with increased risk of uterine, ovarian, and endometrial cancer. For the congenital category, yes, Congential Abnormalities is one major MeSH term represented, and the drugs associated with it do appear to contain some cancer drugs contra-indicated for pregnancy. Fetal Growth Retardation and Microcephaly are also represented. However, the congenital category also catches many generic symptoms that *can* be congenital in nature but in the case of drug side effects are simply referring to symptoms onsetting in adulthood after drug use, such as ichthyosis or dermatitis. The MeSH hierarchy is not perfect, especially since it was not originally designed to capture side effects.

**a) What is the association between the indication that the drug is approved for and having SEs in endocrine/congenital/cardiovascular/oncology/infection?**

Please see Figure S3, which removes the drugs with indications in the same therapy area.

**b) I also don't understand this statement, "Fractions indicate the number of drug-SE pairs with genetic evidence (denominator) and of those, the number that were observed (numerator)." If a drug-SE pair has genetic evidence then by definition wouldn't it have to have been observed? Can you explain in more detail what the numerator and denominator are counting because I don't get it.**

Added: “Throughout, our analysis will use the universe of possible drug-SE pairs, and we will use the term “observed” to refer to those drug-SE pairs where the SE is labeled for the drug.”

**10. In Fig1C are you surprised that OMIM/Genebass evidence provides weaker evidence than GWAS and oncology evidence? This is the reverse of the indication approval evidence. Is this evidence that the drugs aren't hitting the target hard enough or is this somehow related to trait similarity between the SE and the OMIM phenotype?**

**a) are you surprised that germline oncology evidence is so enriched? What genes are represented here? Is there some confounding here?**

For the reader’s benefit we have added some more information to the figure legend: “Note that the germline evidence for oncology is driven by GWAS associations for 5 genes: CYP19A1 (endometrial neoplasms), ESR1 (breast neoplasms), FGFR2 (neoplasms), INSR (polycystic ovary syndrome), and SRD5A2 (breast neoplasms).”

**11. Small omissions and grammar changes:**

**a) First sentence of introduction: Put a metric here, don't make the reader go to the reference to figure it out. If it is major, how major is it?**

Added: “with clinical safety findings accounting for 25% of drug program terminations in Phase I-II”

**b) 4 sentence of the 2nd paragraph of the introduction: "Off-target SEs occur AS the result..." NOT "Off-target SEs occur are the result..."**

Fixed.

**c) 2nd sentence of the 2nd paragraph of the results: I believe a p-value is missing in the parentheses in front of "Fisher's exact test".**

Added: P = 7.1e-93

**The manuscript can be published as is, after correcting minor formatting issues highlight in point 11 and addressing some the ambiguity in definitions in figures, but I believe the impact and insight provided by it will be greatly reduced than if the authors perform the additional analyses suggested. Currently the manuscript is an expansion and replication of previous work and therefore inline with expectation, and leaves the drug developer unsure what to do with the results; which perhaps is fine, just that genetics and pharmacological perturbations of genes provide similar results.**

We have addressed many of the requests above and we agree that this makes for a manuscript that is both more thorough and more readable. We thank the reviewer for this constructive feedback.

**Have all data underlying the figures and results presented in the manuscript been provided?**

**Large-scale datasets should be made available via a public repository as described in the PLOS Genetics data availability policy, and numerical data that underlies graphs or summary statistics should be provided in spreadsheet form as supporting information.**

**Reviewer #1: Yes**

**Reviewer #2: Yes**

**PLOS authors have the option to publish the peer review history of their article (what does this mean?). If published, this will include your full peer review and any attached files.**

**If you choose “no”, your identity will remain anonymous but your review may still be made public.**

**Do you want your identity to be public for this peer review? For information about this choice, including consent withdrawal, please see our Privacy Policy.**

**Reviewer #1: No**

**Reviewer #2: Yes: Aaron G. Day-Williams**
